# Early and Mid-Term Results of Solid Organ Transplantation After Circulatory Death: A 4-Year Single Centre Experience

**DOI:** 10.3390/medicina61122126

**Published:** 2025-11-28

**Authors:** Antonella Galeone, Marilena Casartelli Liviero, Alex Borin, Rostand Emmanuel Nguefouet Momo, Leonardo Gottin, Francesco Onorati, Irene Maffei, Marco Schiavon, Paolo Persona, Tiziano Menon, Luigino Boschiero, Alessandro Antonelli, Giovanni Battista Luciani, Amedeo Carraro

**Affiliations:** 1 Department of Surgery, Dentistry, Pediatrics and Gynecology, Division of Cardiac Surgery, University of Verona, 37129 Verona, Italy; 2 Division of Neurosurgery and Intensive Care Unit, Azienda Ospedaliera Universitaria Integrata of Verona, 37126 Verona, Italy; 3 Liver Transplant Unit, Azienda Ospedaliera Universitaria Integrata of Verona, 37126 Verona, Italyamedeo.carraro@aovr.veneto.it (A.C.); 4 Kidney Transplant Unit, Azienda Ospedaliera Universitaria Integrata of Verona, 37126 Verona, Italy; 5 Department of Surgery, Dentistry, Pediatrics and Gynecology, Division of Anesthesiology, University of Verona, 37129 Verona, Italy; 6 Department of Cardiac, Thoracic, Vascular Sciences and Public Health, Unit of Thoracic Surgery, University of Padova, 35122 Padova, Italy; 7 Department of Surgery, Dentistry, Pediatrics and Gynecology, Division of Urology, University of Verona, 37129 Verona, Italy

**Keywords:** transplantation, donation after circulatory death, normothermic regional perfusion, organ procurement, primary graft dysfunction, primary non-function, delayed graft function

## Abstract

*Background and Objectives*: The use of controlled donation after circulatory death (cDCD) donors has significantly increased during the past decades and successfully expanded the donors’ pool. However, warm ischemia may have detrimental effects on graft function. Italian Law requires a no-touch period of at least 20 min, which is much longer compared to the 5 min accepted in most European countries. *Materials and Methods* This is an Italian single-centre retrospective review of all cDCD procedures performed from April 2021 to June 2025. Patients with severe brain injury undergoing withdrawal of life-sustaining therapy (WLST) were considered for cDCD. After cardiac arrest and a no-touch period of 20 min, organ reperfusion was performed using abdominal or thoraco-abdominal normothermic regional perfusion (NRP) through femoral vessels cannulation. The primary endpoint was 30-day graft survival; secondary endpoints included: incidence of primary non-function (PNF) and non-anastomotic biliary stricture (NAS) in liver transplantation, PNF and delayed graft function (DGF) in kidney transplantation, primary graft dysfunction (PGD) in heart and lung transplantation, and recipient’s survival. *Results*: A total of 52 patients, 33 (63%) males, median age 74 (65–79) years, underwent WLST during the study period and were included in the cDCD program. Median functional warm ischemic time (WIT), total WIT, asystolic phase, and NRP duration were 37 (34–40), 40 (37–42), 24 (23–26), and 192 (166–212) min, respectively. A total of 123 organs (46 livers, 61 kidneys, 8 hearts, and 8 lungs) were considered suitable for transplantation, procured, and successfully transplanted in 115 recipients. We report the early and mid-term outcomes of 84 recipients, including 41 liver recipients, 32 kidney recipients, and 8 heart recipients transplanted at the Azienda Ospedaliera Universitaria Integrata of Verona, and 3 lung recipients transplanted at the Azienda Ospedale Università of Padova. The 30-day graft survival was 95% in liver recipients, 97% in kidney recipients, and 100% in heart and lung recipients. PNF was observed in two liver recipients, and PGD in two lung recipients. DGF was recorded in 3 (9%) kidney recipients. Six recipients died during the follow-up, and the mean survival time was 3.9 ± 0.1 years. *Conclusions*: Solid organ transplantation using cDCD donors is feasible and provides excellent early and mid-term results despite longer donor asystolic times. Larger data and longer follow-up are necessary to confirm these promising results.

## 1. Introduction

Human organ transplantation, including the first heart transplant, developed in the 1960s using donors declared dead with circulatory criteria or non-heart-beating donors [1,2]. After the first publication of the definition of brain death and the criteria to ascertain brain death by the Harvard Medical School in 1968 [3], most organ transplants have been performed with donors declared dead using neurological criteria or heart-beating donors. The limited availability of suitable organs for transplantation has led to the use of expanded criteria donors [4,5,6] and has also renewed interest in donation after circulatory death (DCD) in addition to donation after brain death (DBD). DCD transplant programs are increasing in many European countries [7], and currently DCD donation accounts for more than 50% of all deceased donors in Belgium, the Netherlands, and the United Kingdom [8,9]. DCD donors can be classified into four categories according to the Maastricht classification of 1994 [10], subsequently modified at the sixth International Conference on Organ Donation after Circulatory Death held in Paris in 2013 [11] (Table 1). In countries as Belgium, the Netherlands, Spain, and Canada with a legal framework for medically assisted death, a fifth category also exists for donation after euthanasia [12].

The third category refers to patients with catastrophic cerebral lesions and poor prognosis who do not fulfil all the neurological criteria for brain death diagnosis. In these patients, a withdrawal of life-sustaining therapy (WLST) is planned, which is usually followed by cardiac arrest. In Maastricht III donors, DCD is controlled as the cardiac arrest is awaited. After cardiac arrest occurs, it is mandatory to observe a no-touch period that varies from 2 to 30 min across European countries [7], to ensure that autoresuscitation will not occur [13]. After the declaration of death, it is possible to proceed with surgical techniques aimed at the reperfusion, recovery, and assessment of the abdominal and thoracic organs. One technique to restore organs’ perfusion is the Normothermic Regional Perfusion with an extra-corporeal membrane oxygenation (ECMO) through femoral vessels cannulation, while cerebral reperfusion is prevented by an intra-aortic balloon occlusion. Other techniques include the direct procurement and perfusion (DPP) based on the rapid removal of the organs after declaration of death and the use of ex situ machine perfusion for organ reconditioning. The aim of this study was to evaluate the short and mid-term results of solid organ transplantation using cDCD donors with the use of NRP of an Italian single centre over a 4-year period.

## 2. Materials and Methods

This study was conducted in accordance with the Declaration of Helsinki and approved by the Ethics Committee of the Azienda Ospedaliera Universitaria Integrata (AOUI) of Verona (approval number: IU 301216 06; approval date: 3 September 2018).

This is an Italian single-centre retrospective review of all cDCD procedures performed from April 2021 to June 2025. cDCD was considered in patients with devastating brain injury and poor prognosis who did not fulfil all the neurological criteria for brain death diagnosis and who underwent WLST. Organ donation was discussed with the patient’s family after the WLST decision was made, according to the patient’s wish to donate.

### 2.1. cDCD Procedure

In addition to WSLT, the patient was draped, and femoral sheaths were placed in the femoral artery and vein. A further sheath was placed in the contralateral femoral artery for arterial pressure invasive monitoring and subsequent intra-aortic balloon introduction. The patient was then extubated to begin WSLT. Once inadequate perfusion (systolic arterial pressure < 50 mmHg and/or oxygen saturation < 75%) appeared, heparin (300 UI/kg) was administered. After cardiac arrest occurred, a no-touch period of 20 min was observed according to Italian Law [14,15]. The declaration of death with cardiac criteria requires a continuous electrocardiographic recording showing the absence of any cardiac electrical activity for at least 20 min; in addition, the no-touch period must start after the establishment of cardiac electrical silence, rather than after circulatory arrest (absence of pulse regardless of the cardiac rhythm). After the declaration of death, femoral vessels cannulation was performed using Seldinger’s technique, and organ reperfusion was established using abdominal NRP (A-NRP) with ECMO equipped with a hemadsorption device (CytoSorb^®^, Aferetica, Bologna, Italy). An intra-aortic balloon was inserted and inflated in the descending thoracic aorta below the origin of the subclavian artery to prevent the perfusion of the brain. The correct positioning of the balloon was verified with radiography. In the case of lung procurement, the donor was re-intubated. The patient was then transferred to the operating theatre (OT) for organ evaluation, cold flushing, and procurement. Additional ex situ machine perfusion was used according to the surgeon’s choice for the further evaluation of liver, kidney, and lung function and suitability for transplantation. In case of heart procurement, WLST and thoraco-abdominal (TA)-NRP were both performed in the OT, and TA-NRP was performed using a conventional cardiopulmonary bypass (CPB) pump with a hemadsorption filter mounted on the pump. After declaration of death, NRP was established through femoral vessels cannulation, and an intra-aortic occluding balloon was inserted and inflated in the descending thoracic aorta. Simultaneously, the sternum and the pericardium were opened. Once exposed, the ascending aorta was cross-clamped, and a cold blood cardioplegic solution (Buckberg type C) was infused in the aortic root through a cannula, and a vent was inserted through the right superior pulmonary vein to unload the left chambers. In the meantime, the epiaortic vessels were identified and clamped, followed by deflation of the intra-aortic balloon. Once the cardioplegic solution was completely administered, the aortic cross-clamp was released. After 30 min of reperfusion, an initial attempt was made to wean the donor from CPB; in case of failure, further attempts were made every 30 min for a maximum of 3 h. Cardiac functionality was established according to hemodynamic and electrocardiogram criteria (mean arterial pressure > 50 mm Hg, absence of arrhythmias) and echocardiographic parameters (ejection fraction > 50%, tricuspid annular plane excursion (TAPSE) > 20 mm, no valve abnormalities, no regional alterations of left ventricular wall motion). Once deemed eligible for donation, the heart was stopped conventionally with cold crystalloid cardioplegic solution (Custodiol) and preserved with cold storage.

**Figure 1:**
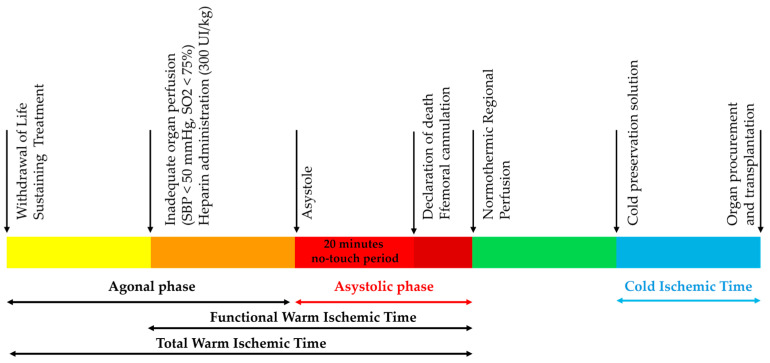
Timeline of the cDCD procedure.

Time elapsed from WSLT (dying patient) to asystole (dead patient) is called the agonal phase and may have a variable duration (Figure 1). If cardiac arrest did not occur within a defined length of time following WLST, typically 90 min, the donation process was stopped, because during this phase, thoraco-abdominal organs are subjected to a warm ischemic time that may damage the organs and compromise their quality. The asystolic phase is defined as the time elapsed from cardiac arrest to NRP initiation and should be <35 min. Functional warm ischemic time (fWIT) was defined as the time elapsed from inadequate organ perfusion to NRP initiation and should be <45 min. Total WIT (tWIT) was defined as the time elapsed from the beginning of the WSLT to NRP initiation and should be <120 min.

NRP duration may vary from 1 to 4 h. Blood samples were collected at baseline before the beginning of the WSLT, after the start of NRP, and every 30 min after the beginning of the NRP for arterial blood gas analysis and to test serum levels of aspartate transaminase (AST), alanine transaminase (ALT), gamma-glutamyl transpeptidase (GGT), creatinine, blood urea nitrogen (BUN), and troponin T in case of heart procurement. Donors were transfused with red blood cells (RBC) as needed to maintain serum levels of hemoglobin above 10 g/dL.

### 2.2. Endpoints

The primary endpoint of the study was to evaluate 30-day graft survival; secondary endpoints included: incidence of primary non-function (PNF) and non-anastomotic biliary stricture in liver transplantation, PNF and delayed graft function (DGF) in kidney transplantation, primary graft dysfunction (PGD) in heart and lung transplantation, and recipient’s survival. Pre-operative characteristics, perioperative data, and in-hospital outcomes were extracted from patients’ paper-based and electronic medical records. Follow-up data were collected until July 2025. The follow-up time was calculated either to death or to the last verified contact with the patient.

### 2.3. Statistical Analysis

Categorical variables are expressed as numbers and percentages, and continuous variables with a skewed distribution are presented as median and interquartile range. The Kaplan–Meier method was used to draw survival curves; the Reverse Kaplan–Meier survival curve was used to calculate the follow-up rate. Statistical analysis has been performed using Sigmaplot version 12.0 (Systat Software Inc., San Jose, CA, USA).

## 3. Results

**Figure 2:**
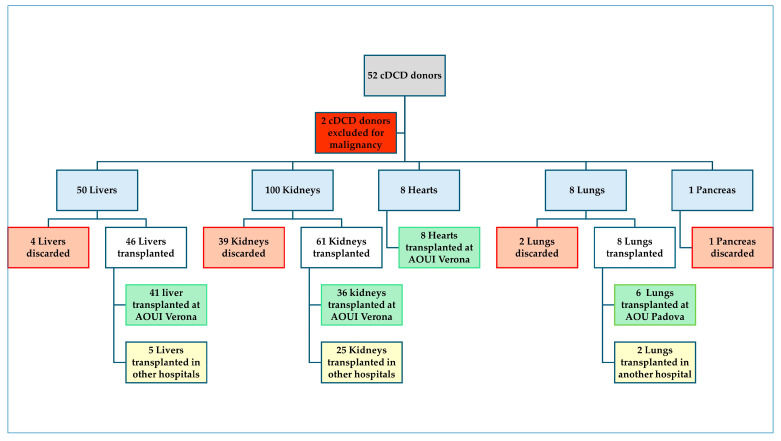
Flowchart of the donation process. AOUI: Azienda Ospedaleria Universitaria Integrata of Verona; AOU: Azienda Ospedale Università of Padova.

From April 2021 to June 2025, a total of 52 patients, 33 (63%) males, median age 74 (65–79) years, underwent WLST and were included in the cDCD program at the AOUI of Verona (Figure 2).

**Figure 3:**
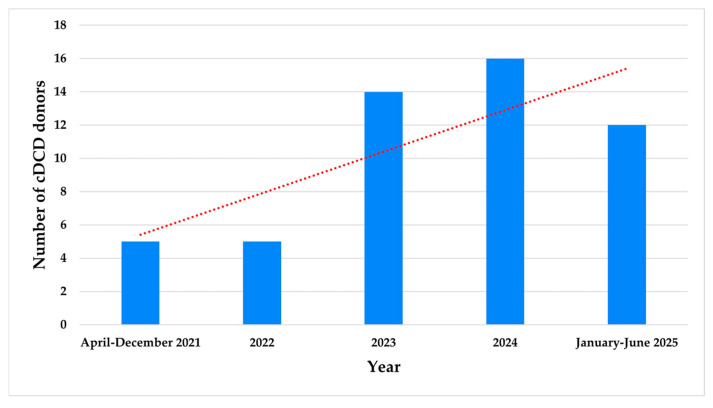
Number of cDCD donors per year from April 2021 to June 2025.

The number of cDCD donors progressively increased during the study period (Figure 3).

**Figure 4:**
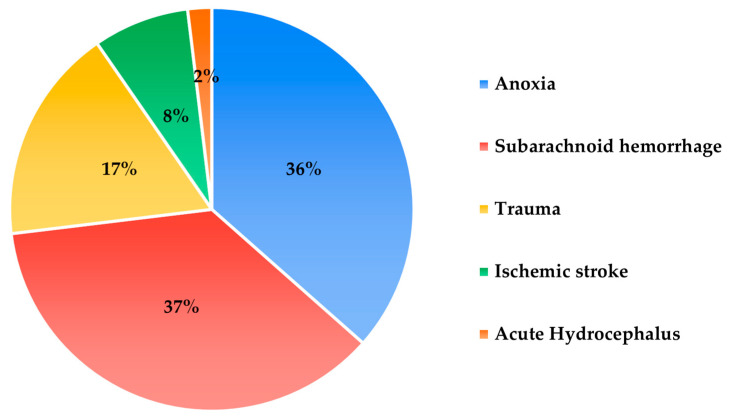
Causes of WSLT.

Causes of WLST included subarachnoid hemorrhage (n = 19, 37%), anoxia (n = 19, 37%), trauma (n = 9, 17%), ischemic stroke (n = 4, 8%), and acute hydrocephalus (n = 1, 2%) (Figure 4).

Donors’ characteristics are illustrated in Table 2.

**Figure 5:**
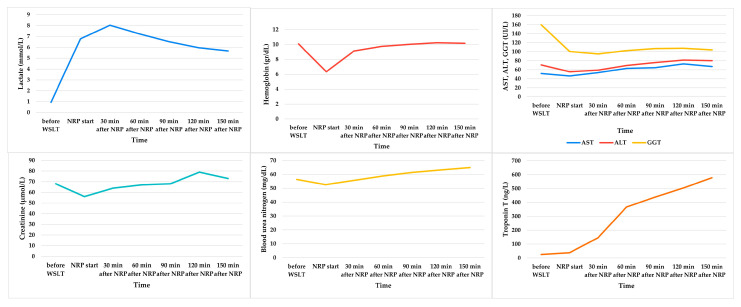
Mean donors’ serum levels of lactate, hemoglobin, AST, ALT, GGT, creatinine, BUN, and Troponin T before WSLT, after the start of the NRP, and every 30 min after the start of the NRP.

All donors who underwent TA-NRP were weaned from CPB at the first attempt. Mean donors’ serum levels of lactate, hemoglobin, AST, ALT, GGT, creatinine, BUN, and Troponin T are depicted in Figure 5.

**Figure 6:**
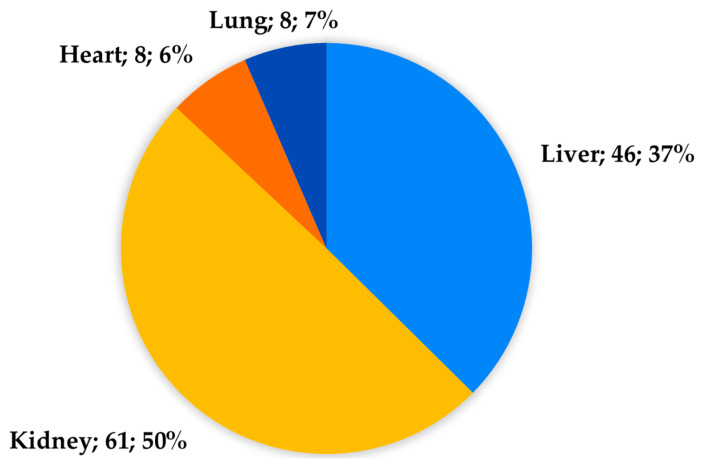
Organs procured and transplanted from cDCD donors.

Two (4%) donors were excluded from organ donation after intra-operative diagnosis of prostatic and pulmonary cancer, respectively. Four livers, thirty-nine kidneys, two lungs, and one pancreas were considered unsuitable for transplantation after NRP initialisation. A total of 123 organs (46 livers, 61 kidneys, 8 hearts, and 8 lungs) were considered suitable for transplantation, procured, and successfully transplanted in 115 recipients (Figure 6).

The early and mid-term outcomes of 84 recipients (41 liver recipients, 32 kidney recipients, and 8 heart recipients transplanted at the AOUI of Verona, and 3 lung recipients transplanted at the Azienda Ospedale Università (AOU) of Padova) are detailed below. The remaining organs were allocated and transplanted in other Italian cities.

### 3.1. Liver Transplantation

A total of 41 patients, 35 (85%) male, median age 58 years, underwent liver transplantation at the AOUI of Verona with an organ from a cDCD donor. Pre, peri, and post-operative recipients’ characteristics are illustrated in Table 3. Of note, ex situ machine perfusion was used in 21 (51%) cases. PNF developed in two (5%) patients; one died, and the other required emergent re-transplantation. Acute cellular rejection grade RAI ≥ 3 was recorded in two recipients, and one of them underwent re-transplantation one year after the first transplant.

### 3.2. Kidney Transplantation

Thirty-two patients, 25 (78%) male, median age 61 years, underwent kidney transplantation at the AOUI of Verona with an organ from a cDCD donor. Pre, peri, and post-operative characteristics are illustrated in Table 4. Ex situ machine perfusion was used in 10 (31%) cases. No case of PNF was recorded. Three (9%) patients had a DGF of 6 ± 1.7 days. One (3%) recipient underwent nephrectomy for a septic complication of the arterial anastomosis 25 days after the kidney transplant.

**Figure 7:**
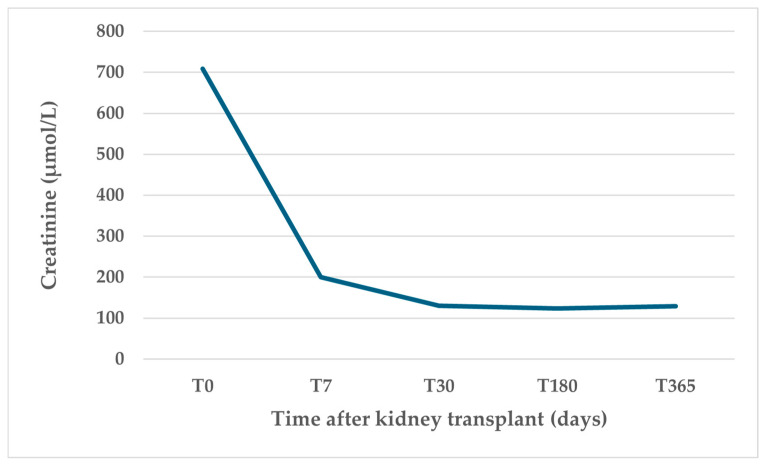
Recipients’ serum levels of creatinine after kidney transplant.

The temporal trend of serum creatinine levels after kidney transplant showed an initial rise followed by a rapid decline 7 days after transplant and stabilization over the subsequent year (Figure 7).

### 3.3. Heart Transplantation

Eight patients, seven (88%) male, median age 63 years, underwent heart transplantation at the AOUI of Verona with an organ from a cDCD donor. Pre, intra, peri, and post-operative characteristics are illustrated in Table 5. Of note, no case of PGD was recorded, and 30-day graft survival was 100%.

### 3.4. Lung Transplantation

Three patients, two (67%) male, median age 64 years, underwent lung transplantation at the AOU of Padova with lungs procured from a cDCD donor using TA-NRP at the AOUI of Verona. Pre, intra, peri, and post-operative characteristics are illustrated in Table 6. EVLP was used in one case for 133 min. PGD ≥ 2 was recorded in two (67%) patients at T0 and T24 and in one (33%) patient at T48 and T72, and 30-day graft survival was 100%.

### 3.5. Overall Recipient’s Survival After Transplantation

Follow-up was 100% complete, and the mean follow-up time was 1.6 ± 0.1 years.

Overall, 30-day graft survival was 96%, as two liver recipients developed PNF (one died 2 days after transplant, and one was retransplanted 2 days after the index liver transplantation), and one kidney recipient developed an infective complication of the arterial anastomosis 25 days after transplant, leading to organ explant.

**Figure 8:**
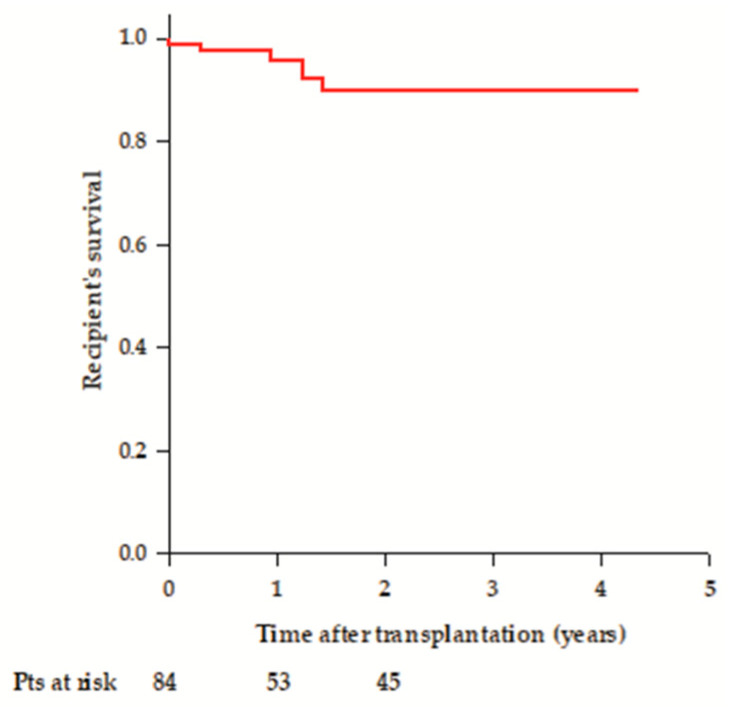
Overall recipient’s survival after transplantation.

Six (7%) recipients died during the follow-up. Causes of death were: PNF, sepsis, pancreatitis, and complications of scleroderma in four liver recipients, pulmonary adenocarcinoma in one kidney recipient, and sepsis in one lung recipient. Mean survival was 4 ± 0.1 years, and survival rates were 98.8% at 30 days, 95.7% at 1 year, and 89.8% at 2 years (Figure 8).

## 4. Discussion

Our series shows that solid organ transplantation using cDCD donors with NRP provides excellent short and mid-term outcomes despite a 20 min no-touch period and longer fWIT, thus implying a constant increase in the use of cDCD donors over time. NRP is mandatory in Italy, but a progressive increase in NRP use has also been registered over time in other countries because of the positive outcomes associated with this technique. In liver transplantation using cDCD donors, concerns related to warm ischemia and damage to the biliary tree have been described; however, the introduction of NRP has considerably improved outcomes [16]. In Spain, organs from cDCD donors have been traditionally recovered with rapid in situ cold preservation to prevent warm ischemic injury as quickly as possible, but actually, NRP is currently far more frequent than super rapid recovery (SRR) for liver procurement. A propensity-score matching (PSM) Spanish nationwide observational cohort study performed using 117 cDCD livers recovered with SRR and 95 recovered with A-NRP from 2012 to 2016, showed that the use of A-NRP reduces rates of overall post-transplant biliary complications, ischemic type biliary lesions, and graft loss and allows the use of livers even from older cDCD donors [17]. A recent publication on more than 800 cDCD liver transplants, the great majority with A-NRP, confirmed the superiority of A-NRP over SRR with respect to biliary complications and recipient’s survival [18]. Accordingly, reports from other European and non-European countries stated that the superiority of NRP for organ recovery from cDCD donors [19,20,21]. A retrospective analysis comparing 43 NRP cDCD donor livers with 187 non-NRP cDCD donor livers transplanted in the UK showed that the use of NRP was associated with a reduction in early allograft dysfunction, 30-day graft loss, freedom from ischemic cholangiopathy, and fewer anastomotic strictures [19]. In the US, where only 11.4% of liver recipients receive a cDCD liver, a retrospective, observational cohort study compared liver transplant outcomes from 242 cDCD donors recovered by NRP (n = 106) vs. SRR (n = 136) and found comparable patient and graft survival in liver transplant recipients of cDCD donors recovered by NRP vs. SRR, with reduced rates of ischemic cholangiopathy, biliary complications, and early allograft dysfunction in NRP recipients [21]. A meta-analysis including 11 studies comparing NRP cDCD livers with both non-NRP cDCD livers and DBD livers showed that NRP is unanimously associated with lower rates of ischemic cholangiopathy, PNF, hepatic artery thrombosis, and other biliary complications and lower rates of recipient death and graft loss compared to non-NRP recovery [22]. Additionally, there was no difference in outcomes between NRP cDCD donation compared to DBD liver donation, and NRP utilization is often associated with more liberal organ acceptance criteria in terms of donor age, agonal time, and graft steatosis, with a discard rate after NRP initialisation of 30% [22]. The discard rates are highest in the Netherlands, UK, and the US, ranging between 70 and 80%, and lower in Belgium, France, Italy, Spain, and Switzerland, ranging between 30 and 40% [23]. Huge differences also exist in the use of machine perfusion, as well as in graft and donor risk factors. The median donor age and functional donor warm ischemia time are highest in Italy (>40 min), followed by Switzerland, France, and the Netherlands. However, differences in risk profiles of accepted DCD livers between countries did not translate into significant differences in 5-year graft survival rates, ranging between 60 and 82% [23]. Countries where in situ and ex situ machine perfusion strategies are used routinely have better DCD utilization rates [23]. In our series, the discard rate of cDCD liver grafts was only 8% despite the use of older donors with a median age of 74 years. Although transaminase release is a widely accepted marker of liver injury, its cut-off has been modified from initially 3–4 times the normal values to upper thresholds reported in the most recent series [19,24]. Lactate clearance has been proposed as a parameter to assess liver function, with a downward lactate trend indicating a well-functioning liver, as in NMP [25]. In our series, serum levels of lactate reached a peak 30 min after NRP initiation and then progressively declined 1 h after NRP initiation. Ex situ machine perfusion after NRP was performed in 51% of cDCD liver grafts with either DHOPE (41%) or NMP (10%). We recorded PNF in 5% of the recipients, which is consistent with previously published studies reporting an incidence of PNF in cDCD liver transplantation ranging from 0% to 5% [18,24,26]. Similarly, the incidence of NAS in our series was 5%, a finding that is comparable to other reports that described this complication in 0 to 5% of the recipients [18,24,26]. A multicentre retrospective Italian cohort study conducted on 44 cDCD donors undergoing NRP showed that DHOPE was used in 84% of cases, and the PNF rate was 5% [24]. A subgroup of 37 cDCD livers, preserved with NRP and D-HOPE, was matched and compared with static-preserved controlled DCD transplants from an established European program. The Italian NRP + DHOPE group showed no statistically significant differences in ischemic cholangiopathy (97% versus 92%), despite the significantly longer donor warm ischemia in Italy (40 versus 18 min) [24]. The implementation of sequential NRP and end-ischemic ex situ machine perfusion may also be useful to expand the donor pool, allowing the safe use of very old cDCD donors. A recent study was conducted on 17 cDCD donors older than 70 years (median age 82 years) who were evaluated during NRP and then randomly assigned to DHOPE or NMP [27]. Six (35%) grafts were not considered suitable for liver transplantation, whereas 11 (65%) were randomly assigned to ex situ DHOPE (n = 6, 55%) and NMP (n = 5, 45%). None was discarded during MP. There were no cases of PNF, one case of postreperfusion syndrome (9%), and two cases (18%) of early allograft dysfunction. At a median follow-up of 8 months, the authors reported no vascular complications or ischemic cholangiopathy and no differences in terms of postoperative hospitalization or complications based on the type of MP [27].

A few clinical series have been published on kidney transplantation using cDCD donors with NRP. Early experience of a Norwegian centre showed no differences in DGF and 1-year graft survival from 14 cDCD kidneys recovered with NRP compared with 163 transplants from DBD donors [28]. Similarly, the first Spanish series of cDCD donors using A-NRP reported no difference in DGF and kidney graft survival from cDCD donors compared with DBD donors [29]. Another Spanish report on 182 kidney recipients, 98 from DBD donors and 84 from cDCD donors, showed that in cDCD donors, the use of NRP had lower rates of PNF and DGF compared with the use of SRR [30]. Additionally, no differences were found in graft survival rates at 1 year, and patient survival rates were >90% in all groups [30]. A retrospective study on cDCD kidneys obtained with NRP (n = 865) and SRR (n = 1437) showed on two cohorts with a total of 770 patients obtained after PSM, no statistically significant differences between the groups in terms of PNF and 1-year mortality; however, the use of SRR was associated with a significantly increased odds of DGF and 1-year graft loss [31]. A large French national cohort study showed that early outcomes of cDCD kidney transplants were better than those of comparable DBD transplants. Using a PSM analysis, the authors observed a significantly lower adjusted risk ratio of DGF occurrence in cDCD than DBD transplants (20% vs. 28%, respectively), while the 1-year graft survival rate was similar in both groups [32]. The authors ascribed the low rate of DGF in cDCD recipients mainly to NRP use and the use of hypothermic machine perfusion, which is mandatory in the French protocol. An Italian preliminary report with sequential NRP and HOPE in 10 kidney transplants from cDCD showed a 30% incidence of DGF and no correlation with creatinine or lactate values during NRP. However, lactate levels in the HOPE perfusate were significantly higher in those cases developing DGF [33]. In our series, ex situ machine perfusion was used only in 31% of the cases; however, there was no PNF, and the DGF rate was 9%, which is both inferior to previously published studies reporting a PNF rate up to 5% and DGF ranging from 7% to 36% [29,30,31,32,33].

The worldwide increasing use of cDCD heart donors during the past decades has successfully permitted the expansion of the donors’ pool and reduced waiting list mortality. The adoption of a DCD heart transplant program has the potential to increase the volume of adult heart transplantation by approximately 15–30% and to reduce the mortality on the waiting list by 40% [34,35]. Concerns about the detrimental effects on graft function induced by warm ischemia have initially slowed down the spread of cDCD heart donation in Italy, where a longer no-touch period is required compared to the 5 min accepted in most European countries [7]. Additionally, in addition to the absence of any cardiac electrical activity for at least 20 min, the no-touch period in Italy must start after the establishment of the electrical asystole that typically occurs several minutes after the mechanical asystole. Both these requirements may significantly prolong the ischemic time and have a negative impact on graft function. The longer length of the no-touch period directly influences the acceptable duration of fWIT, that is, 45 min in Italy, while acceptable fWIT is usually limited to 30 min in most countries. An experimental study aimed to determine the critical warm ischemia time based on in vivo biochemical changes in 16 DCD non-cardiac donors. Serial endomyocardial biopsies were performed immediately before WLST, at circulatory arrest, and every 2 min thereafter, to assess calcium homeostasis, mitochondrial function, and cellular viability. Compared to baseline, no significant deterioration was observed in any studied parameter at the time of cardiac arrest. However, beyond 10 min after cardiac arrest, a reduction in contractility and mitochondrial activity and an increase in apoptosis were observed, indicating a significant compromise in cellular function and viability [36]. Despite these premises, cDCD heart transplantation began in May 2023 in Italy, and preliminary results of a multicentre study showed that heart transplantation from cDCD donors is feasible even with longer fWIT, showing 100% 30-day graft survival, 90.5% 30-day patient survival, and 23% severe PGD rate [37]. In our series, we did not observe any case of PGD, and both 30-day graft survival and recipients’ survival at the end of the follow-up were 100%. A proper donor selection, colocalization of donor and recipient, and shorter cold ischemic times may have contributed to these better results. In situ resuscitation of the heart with TA-NRP was pioneered in the UK by the Royal Papworth Hospital in 2015 [38]. Previous studies from both Europe and the US found no differences in short and long-term outcomes between cardiac allografts from TA-NRP DCD compared with DBD donors [39,40,41,42]. However, this technique continues to raise ethical concerns due to the risk of brain perfusion via collateral circulations to the vertebrobasilar system during TA-NRP, despite the clamping of supra-aortic trunks, which could invalidate the diagnosis of death [43]. Other reports demonstrated that cDCD with TA-NRP did not restore brain blood flow [44], but these are limited by small numbers; therefore, a larger cohort and a global collaboration have been advocated to definitively establish the absence of brain blood flow or perfusion [45]. Another technique known as direct procurement and preservation (DPP) has been proposed to recover the heart ex situ with the use of NMP by Dhital and coll. [46]. The review of the first 8 years of cDCD heart transplant activity from St Vincent’s in Australia using the DPP technique demonstrated a significant reduction in the incidence of severe PGD from 35% to 8%. One- and five-year survival of cDCD heart transplant recipients were comparable to that of a contemporary cohort of DBD recipients [47]. Few studies have compared outcomes after TA-NRP and DPP. Some studies reported no difference in the recipient’s survival between TA-NRP and DPP techniques for heart recovery after cDCD heart transplant [48,49]. An analysis of the United Network for Organ Sharing (UNOS) database demonstrated similar rates of PGD between TA-NRP and DPP, with a severe PGD rate of 9.4% and 9.7%, respectively [50]. However, a recent international study of 504 cDCD heart transplants from 22 centers in 4 countries showed better outcomes with TA-NRP compared to DPP [51]. TA-NRP was associated with a 60% reduction in the incidence of severe PGD and a 40% reduction in the incidence of acute cellular rejection requiring treatment compared to DPP, despite similar donor and recipient demographics in both TA-NRP and DPP groups [51].

When both the heart and other thoracic and abdominal organs are procured from a cDCD donor, questions remain about the potential impact of heart procurement strategies on thoraco-abdominal organ utilization and outcomes. In addition to offering a cheaper alternative to organ recovery than DPP, there is a growing body of evidence that TA-NRP is associated with better utilization of thoracic and abdominal organs from the donor and with superior recipient outcomes [52]. The transplantation rate of concurrently procured cDCD livers was higher with TA-NRP compared to direct procurement. There was no difference in the 6-month liver graft failure rate. Recipients of kidneys procured with in situ perfusion had less DGF, shorter length of stay, and lower serum creatinine at discharge. Six-month recipient survival in the direct procurement and in situ perfusion group was similar after cDCD liver and kidney transplantation [53]. Previous studies also raised concerns about the potential of TA-NRP to compromise the quality and utilization of lung allografts by inducing inflammation-mediated lung injury, pulmonary oedema, and hampering lung function assessment [54,55]. A recent report on 85 DBD and 23 cDCD TA-NRP lung transplants suggested that TA-NRP does not negatively affect lung allografts during procurement. PGD grade 3 rates at postoperative day 0, 1, 2, and 3, and overall survival were not significantly different between cDCD and DBD recipients [56]. An analysis of the UNOS database on 627 cDCD donors whose hearts were procured (211 in situ perfused, 416 directly procured) showed no difference in lung utilization rates between in situ perfused donors (14.9%) and directly procured donors (13.8%). Following transplantation, lung recipients from in situ perfused donors required numerically lower rates of ECMO and mechanical ventilation at 72 h. Six-month post-transplant survival was similar between groups [57]. Similarly, other reports found no difference in lung utilization [51] and recipients’ outcomes [58,59] between TA-NRP and DPP. Lower rates of PGD in lungs recovered using TA-NRP compared to those recovered during A-NRP have been reported [60].

We acknowledge that this study has some limitations due to its retrospective design and nature, the small sample size, the lack of a control group, and the short follow-up period. Therefore, the results of this series should be interpreted with caution.

## 5. Conclusions

Solid organ transplantation using cDCD donors recovered with NRP provides excellent early and mid-term results despite longer donor asystolic times. A proper donor selection and the co-localization of the potential donor to the recipient transplant center may have contributed to the positive results obtained in this series due to the reduction in cold ischemic time. These promising results need to be confirmed with larger data and longer follow-up. However, this series provides further evidence supporting the continued use of NRP in cDCD solid organ transplantation, as it improves the recovery, assessment, quality, and utilization rates of cDCD organs. A more widespread adoption of cDCD with TA-NRP may contribute to increasing the donor pool, limiting organ shortage, reducing mortality on the waiting list, and also expanding indications for transplantation.

## Figures and Tables

**Table 1:** Modified Maastricht classification of DCD donors.

Category	Description	Type
I Ia: out-of-hospital Ib: in-hospital	Unwitnessed cardiac arrest (Dead on arrival/Found dead)	Uncontrolled
II IIa: out-of-hospital IIb: in-hospital	Witnessed cardiac arrest (Unsuccessful resuscitation)	Uncontrolled
III	Awaiting cardiac arrest after WSLT	Controlled
IV	Cardiac arrest while brain dead	Uncontrolled/ controlled
V	Medically assisted cardiac arrest (Euthanasia)	Controlled

**Table 2:** Donors’ characteristics.

**Donors’ Characteristics**	**n = 52**
Sex, male	33 (63%)
Age, years	74 (65–79)
BMI	25 (23–29)
BSA	1.8 (1.7–1.9)
Agonal phase, min	13 (10–15)
fWIT, min	37 (34–40)
tWIT, min	40 (37–42)
Asystolic phase, min	24 (23–26)
NRP, min	192 (166–212)
A-NRP	44 (85%)
TA-NRP	8 (15%)

A-NRP: abdominal normothermic regional perfusion. BMI: body mass index. BSA: body surface area. fWIT: functional warm ischemic time. NRP: normothermic regional perfusion. TA-NRP: thoraco-abdominal normothermic regional perfusion. tWIT: total warm ischemic time.

**Table 3:** Liver recipients’ pre, peri, and post-operative characteristics.

Recipients’ Characteristics	Liver Recipient (n = 41)
*Preoperative characteristics*	
Age, years	58 (51–65)
Sex, male	35 (85%)
BMI	25 (23–28)
Liver disease: Alcoholic HCV infection HBV infection Autoimmune Biliary atresia Cholestasis	19 (46%) 10 (24%) 6 (15%) 4 (10%) 1 (2%) 1 (2%)
Hepatocarcinoma	21 (51%)
Portal vein thrombosis	3 (7%)
MELD score	14 (11–26)
MELD Na score	17 (11–26)
Renal replacement therapy	5 (12%)
Cardio-respiratory support	6 (15%)
*Peri and post-operative characteristics*	
Cold Ischemia, min	240 (179–320)
Ex situ machine perfusion: DHOPE NMP	21 (51%) 17 (41%) 4 (10%)
Primary non-function	2 (5%)
ICU stay, days	2 (1–4)
Hospital stay, days	18 (13–29)
Hepatic artery thrombosis	1 (2%)
Portal vein thrombosis	1 (2%)
Biliary leak	6 (15%)
Anastomotic biliary stricture	7 (17%)
Non-anastomotic biliary stricture	2 (5%)
Ischemic cholangiopathy	0
Acute cellular rejection RAI ≥ 3	2 (5%)
Re liver transplant	2 (5%)
30-day graft survival	39 (95%)
30-day patient survival	40 (98%)

**Table 4:** Kidney recipients’ pre, peri, and post-operative characteristics.

Recipients’ Characteristics	Kidney Recipient (n = 32)
*Preoperative characteristics*	
Age, years	61 (54–68)
Sex, male	25 (78%)
BMI	25 (23–28)
Kidney disease: ADPKD Chronic glomerulonephritis Nephroangiosclerosis Membranous glomerulonephritis Focal segmental glomerulosclerosis Congenital single kidney IgA nephropathy Membranoproliferative glomerulonephritis Diabetic nephropathy Balkan endemic nephropathy SLE Wegener granulomatosis Vesicoureteral Reflux Obstructive uropathy	8 (25%) 6 (17%) 4 (13%) 2 (6%) 2 (6%) 2 (6%) 1 (3%) 1 (3%) 1 (3%) 1 (3%) 1 (3%) 1 (3%) 1 (3%) 1 (3%)
Dialysis: Hemodialysis Peritoneal dialysis	18 (56%) 11 (34%)
Pre-emptive kidney transplant	3 (9%)
*Peri and post-operative characteristics*	
Ex situ machine perfusion	10 (31%)
Cold ischemic time, hours	6 (5–7)
Double kidney transplant	4 (13%)
Primary non-function	0
Delayed graft function	3 (9%)
Acute cellular rejection	0
Re-transplant	0
30-day graft survival	31 (97%)
30-day patient survival	32 (100%)

**Table 5:** Heart recipients’ pre, peri, intra, and post-operative characteristics.

Recipients’ Characteristics	Heart Recipients (n = 8)
*Pre-operative characteristics*	
Age, years	63 (58–68)
Sex, male	7 (88%)
BMI	26 (23–32)
BSA, m^2^	2 (1.8–2.1)
Cardiomyopathy: Dilatative Ischemic Valvular Endocardial fibroelastosis	3 (38%) 3 (38%) 1 (12%) 1 (12%)
Diabetes	3 (38%)
Pulmonary hypertension	1 (12%)
Redo surgery	2 (25%)
LVAD	1 (12%)
Status at transplant: 2A 2B	2 (25%) 6 (75%)
Waiting list time, days	190 (39–615)
*Intra and peri-operative* *characteristics*	
Cold ischemic time, min	76 (67–84)
CPB, min	166 (130–200)
Primary graft dysfunction	0
ECMO/IABP	0
Lactate peak, mmol/L	7 (4–13)
Troponin T peak, ng/L	2389 (1804–2770)
CRRT	2 (25%)
Mechanical ventilation, days	4 (2–5)
ICU stay, days	7 (5–11)
Hospital stay, days	32 (24–34)
*Post-operative characteristics*	
Acute cellular rejection grade ≥ 2R	2 (25%)
Cardiac allograft vasculopathy	1 (12%)
LVEF at last follow-up, %	60 (57–60)
Re-transplant	0
30-day graft survival	8 (100%)
30-day patient survival	8 (100%)

**Table 6:** Lung recipients’ pre, peri, intra, and post-operative characteristics.

Recipients’ Characteristics	Lung Recipients (n = 3)
*Pre-operative characteristics*	
Age, years	64 (64–65)
Sex, male	2 (67%)
Lung disease: CPFE Emphysema ILD	1 (33%) 1 (33%) 1 (33%)
LAS	37 (33–38)
Hospitalization	0
Mechanical ventilation	0
ECMO	0
Waiting list time, days	5 (19–60)
*Intra and peri-operative characteristics*	
EVLP	1 (33%)
Cold ischemia, min Right lung Left lung	275 (272–317) 190 (183–240)
Intra-operative ECMO time, min	188 (163–208)
Bilateral lung transplant	3 (100%)
Primary graft dysfunction ≥ 2: T0 T24 T48 T72	2 (67%) 2 (67%) 1 (33%) 1 (33%)
Mechanical ventilation, hours	24 (12–42)
ICU stay, days	6 (5–6)
Hospital stay, days	30 (29–38)
*Post-operative characteristics*	
Bronchial stenosis	1 (33%)
Acute cellular rejection	0
CLAD	0
Re-transplant	0
30-day graft survival	3 (100%)
30-day patient survival	3 (100%)

## Data Availability

The data presented in this study are available upon request from the corresponding author.

## Abbreviations

The following abbreviations are used in this manuscript:

ADPKD	Autosomal Dominant Polycystic Kidney Disease
ALT	Alanine transaminase
AOUI	Azienda Ospedaliera Universitaria Integrata of Verona
AST	Aspartate transaminase
BMI	Body Mass Index
cDCD	Controlled Donation after Circulatory Death
CLAD	Chronic Lung Allograft Dysfunction
CPB	Cardio-pulmonary bypass
CPFE	Combined pulmonary fibrosis and emphysema
CRRT	Continuous Renal Replacement Therapy
D-HOPE	Dual Hypothermic oxygenated machine perfusion
ECMO	Extra-corporeal membrane oxygenation
EVLP	Ex vivo lung perfusion
GGT	Gamma-glutamyl transpeptidase
ICU	Intensive Care Unit
ILD	Interstitial Lung Disease
LAS	Lung allocation score
NMP	Normothermic machine perfusion
NRP	Normothermic Regional Perfusion
PSM	Propensity score matching
RAI	Rejection Activity Index
SLE	Systemic lupus erythematosus
UNOS	United Network for Organ Sharing
WIT	Warm Ischemic Time
WLST	Withdrawal of Life Sustaining Therapy
